# Two cyclometalated Pt(ii) complexes showing reversible phosphorescence switching due to grinding-induced destruction and crystallization-induced formation of supramolecular dimer structure†

**DOI:** 10.1039/d1ra07142d

**Published:** 2021-12-21

**Authors:** Qin-Zhen Yuan, Fu-Shun Wan, Ting-Ting Shen, Deng-Ke Cao

**Affiliations:** State Key Laboratory of Coordination Chemistry, School of Chemistry and Chemical Engineering, Nanjing University Nanjing 210093 P. R. China dkcao@nju.edu.cn

## Abstract

Complexes [Pt(dfppy)(pbdtmi)]PF_6_ (1) and [Pt(ppy)(pbdtmi)]PF_6_ (2) have been constructed based on dithienylethene-based N^N ligand pbdtmi, showing supramolecular dimer structure in which two coordination cations connect each other through π⋯π stacking interaction. The crystalline state samples of both 1 and 2 reveal strong phosphorescence (emission peak: around 579 nm for 1, and 551 nm for 2). Interestingly, a grinding treatment for either 1 or 2 leads to phosphorescence switching from on-state to off-state. The subsequent crystallization with toluene recovers the initial on-state. This work discusses the relationship between the supramolecular dimer structures and the related phosphorescence switching behaviors in 1 and 2, and also explores the photochromism of pbdtmi, 1 and 2.

## Abstract 2

Two Pt(ii) complexes 1 and 2 reveal similar supramolecular dimer structure, in which two [Pt(dfppy/ppy)(pbdtmi)]^+^ cations connect each other through the π⋯π stacking interaction. Thus these complexes show reversible phosphorescence switching by grinding and crystallization with toluene.

## Graphical abstract

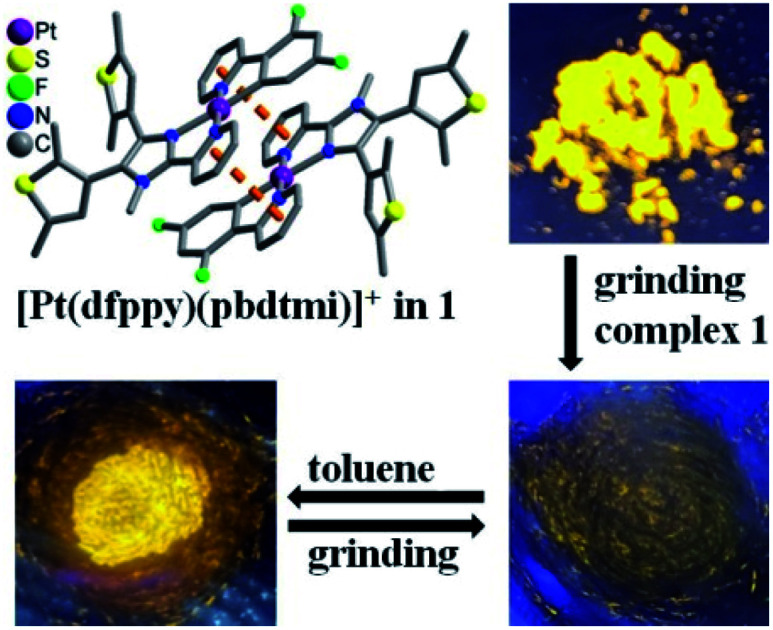

## Introduction

Cyclometalated Pt(ii) complexes have attracted considerable attention due to their luminescence with potential applications in luminescence switching materials,^1,2^ sensing,^3,4^ bioimaging,^5,6^ and organic light-emitting diodes (OLEDs).^7,8^ In order to modulate the structures and the related luminescence of Pt(ii) complexes, one effective strategy is to incorporate various cyclometalated ligands (*e.g.* 2-phenyl-pyridine and its derivatives) and/or ancillary ligands L^X.^9,10^ The other is to control intermolecular interactions, such as Pt⋯Pt interaction,^11,12^ hydrogen bond,^13^ and π⋯π stacking interaction,^14,15^ which can significantly affect the luminescence of Pt(ii) complexes.

It is well known that dithienylethene (DTE) is a class of fascinating organic compounds, showing photochromism due to light-irradiation-induced structural transition between open form and closed form (Scheme S1†).^16^ So far, some DTE-based Pt(ii) complexes have been reported, in which various DTE ligands coordinate with Pt(ii) ions through some functional groups such as alkynyl group,^17–21^ pyridyl group,^22^ 1,10-phenanthroline unit,^23^ 2-(thiophen-2-yl)-pyridine moiety,^24^ 2-(2-pyridyl)imidazole moiety,^25^ and 1,3-di(2-pyridyl)benzene unit.^26^ Some complexes were found to exhibit phtochromism, and even significant switching of either luminescence or nonlinear optical (NLO) property due to the open/closed isomerization of DTE unit.^18,22,23,25,26^ However, no document has so far reported a DTE-based Pt(ii) complex showing supramolecular structure and the related functional property.

**Scheme 1:**
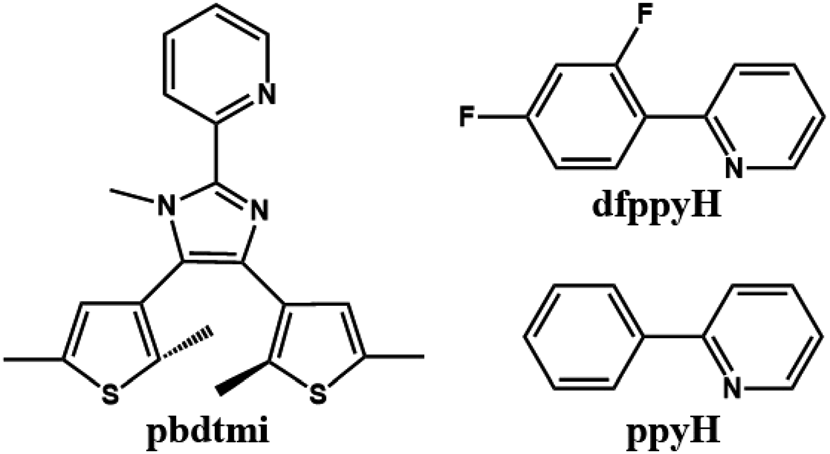
Molecular structures of pbdtmi, dfppyH and ppyH.

Based on DTE-based N^N ancillary ligands (Scheme S2†), some alkynylplatinum(ii) diimine complexes have been reported, in which the molecular structures of these DTE-bsed N^N ligands can significantly modulate the luminescence and the photochromism of the related Pt(ii) complexes in solution.^23,25^ Inspired by these studies, we synthesized a new DTE-based N^N ancillary ligand 2-pyridyl-4,5-bis(2,5-dimethyl(3-thienyl))-1-methyl-imidazole (pbdtmi, see Scheme 1) and its cyclometalated Pt(ii) complexes [Pt(dfppy)(pbdtmi)]PF_6_ (1) and [Pt(ppy)(pbdtmi)]PF_6_ (2) in which dfppyH = 2-(2,4-difluorophenyl)-pyridine, and ppyH = 2-phenyl-pyridine (Scheme 1). Besides possible photochromism, ligand pbdtmi has three structure advantages. (1) Its pyridine–imidazole moiety can readily coordinate to a {Pt(dfppy/ppy)}^+^ unit, forming the corresponding cyclometalated Pt(ii) complex. (2) The imidazole unit in ligand pbdtmi connects a CH_3_ group, thus avoiding the formation of hydrogen bonds among neighboring molecules in complexes 1 and 2, and also facilitating the formation of inter-molecular π⋯π stacking interaction. (3) The two 2,5-dimethylthiophene groups in ligand pbdtmi not only can improve the solubility of 1 and 2, but also can provide suitable steric hindrance to modulate molecular packing structure. Herein, we report the syntheses and crystal structures of complexes 1 and 2, and discuss their reversible phosphorescence switching behaviors and photochromism.

## Results and discussion

### Syntheses and structural characterizations

Complexes 1 and 2 were readily synthesized through the reaction of pbdtmi, [Pt(dfppy)(SEt_2_)Cl]/[Pt(ppy)(SEt_2_)Cl] and AgPF_6_ in CH_2_Cl_2_ at 40 °C for one day. The two complexes can also be prepared through the reaction of pbdtmi and the corresponding dichloride bridged dimer [Pt(dfppy)Cl]_2_/[Pt(ppy)Cl]_2_, and the following anion exchange of Cl^−^ with PF_6_^−^. However, this synthesis approach needs to use tedious silica column chromatography to purify the crude product. The structures of 1 and 2 were characterized by ^1^H NMR, IR spectra, elemental analyses, and crystal structures.

### Crystal structures of 1 and 2

**Fig. 1:**
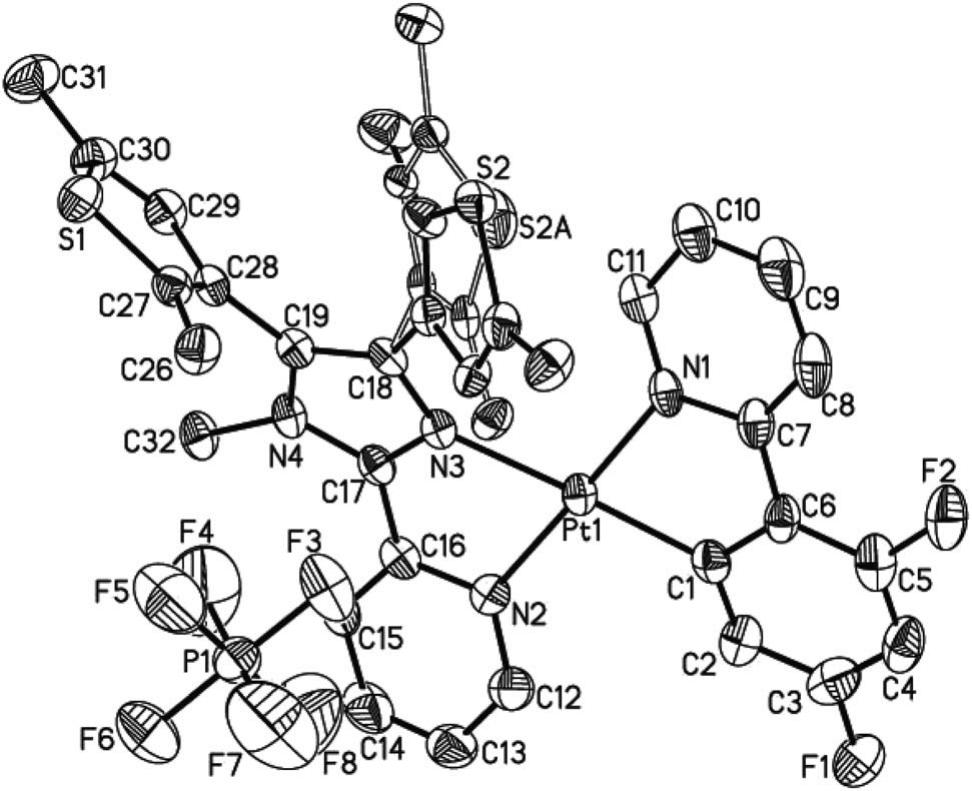
Thermal ellipsoid plot (50% probability) of the molecular structure of 1. All H atoms are omitted, and the atom labels of the disordered thiophene group were omitted for clarity.

The single crystals of both 1 and 2·C_7_H_8_ (C_7_H_8_ = toluene) were grown in a CH_2_Cl_2_–toluene mixture, and measured by X-ray crystallography. The crystallographic data, the selected bond lengths and bond angles are summarized in Tables S1–S3 (ESI†). Complex 1 and 2·C_7_H_8_ crystallize in the monoclinic space group *P*2_1_/*n* and *P*2_1_/*c*, respectively, showing similar molecular structures (Fig. 1 and S5†). A neutral N^N ligand pbdtmi coordinates to a {Pt(dfppy)}^+^ unit in complex 1, and a {Pt(ppy)}^+^ unit in complex 2. The resultant [Pt(dfppy/ppy)(pbdtmi)]^+^ cation and its counter ion PF_6_^−^ are held together by electrostatic interaction. Each Pt(ii) ion in 1 and 2 shows a distorted square planar geometry. Two of the four coordination sites are occupied by the C1 and N1 atoms from a dfppy^−^/ppy^−^ ligand. The remaining two coordination sites are occupied by pyridine nitrogen N2 and imidazole nitrogen N3 from a pbdtmi ligand. The dihedral angle between N1–Pt1–C1 plane and N2–Pt1–N3 plane is 3.5(1)° in complex 1, and 2.9(1)° in complex 2. The coordination of dfppy^−^/ppy^−^ and pbdtmi towards the Pt(ii) ion results in the formation of two five-membered rings (Fig. 1 and S5†). Thus, the bond angles N1–Pt1–C1 and N2–Pt1–N3 [79.75(19)° and 77.88(15)° in 1; 79.8(2)° and 77.39(17)° in 2] notably deviate from ideal 90°. Due to these steric constraints, the other two bond angles N1–Pt1–N3 and C1–Pt1–N2 [101.14(16)° and 101.30(18)° in 1; 101.76(18)° and 101.0(2)° in 2] are significantly larger than 90°. Around a Pt(ii) ion in the molecular structure of either 1 or 2, the Pt1–N3 bond length [2.111(4) Å in 1, 2.124(4) Å in 2] is significantly longer than the others Pt1–C(N) distances [2.002(5)–2.050(4) Å in 1 and 2]. This can be due to the fact that the N3 atom is *trans* to the σ-bound C1 atom (Fig. 1 and S5†).^27^

**Fig. 2:**
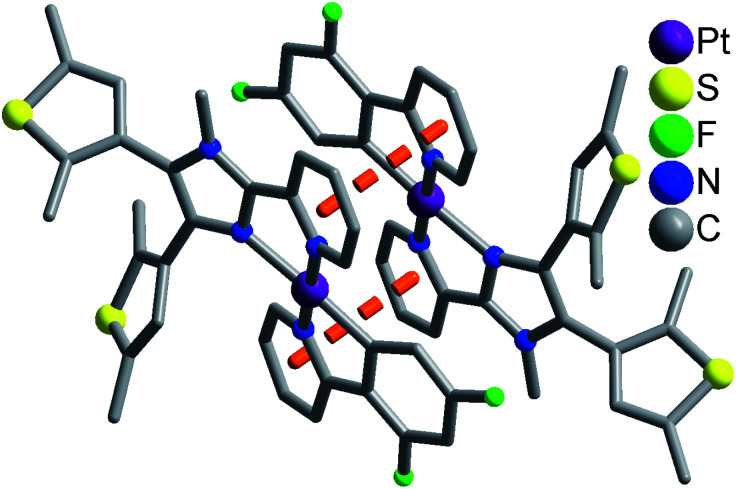
Supramolecular dimer structure in 1. The disordered state of a thiophene group has not been shown for clarity.

Complexes 1 and 2 exhibit similar stacking structures (Fig. 2 and S6†). Two neighboring [Pt(dfppy/ppy)(pbdtmi)]^+^ cations in these complexes connect each other through the π⋯π stacking interaction between two pyridine rings from dfppy^−^/ppy^−^ ligand and pbdtmi ligand [centroid–centroid distance: 3.544(1) Å in 1, and 3.595(1) Å in 2], thus forming supramolecular dimer structures (Fig. 2 and S6†). The Pt⋯Pt distance is 3.627(1) Å in 1, and 3.698(1) Å in 2, hence there is no Pt⋯Pt interaction.^28,29^ These dimers stack through van der Waals interactions, and the inter-dimer space is filled with PF_6_^−^ anions and/or toluene molecules (Fig. S7 and S9†).

### Electronic absorption spectra

Complexes 1 and 2 in CH_2_Cl_2_ have similar absorption spectra (Fig. S9 and Table S4†), showing two strong bands (around 306 and 359 nm for 1, and around 310 and 363 nm for 2) and a weak absorption tail (toward 448 nm for 1, and toward 456 nm for 2). In contrast, compound pbdtmi only exhibits a broad band at 320 nm. For complexes 1 and 2, the absorption bands at 306 nm and 310 nm can be assigned to ligand-centered (^1^LC) transitions (dfppy^−^/ppy and pbdtmi ligands), while the bands around 359 nm and 363 nm should be a combination of metal-to-ligand charge transfer (^1^MLCT) and ligand-centered (^1^LC) transitions, because of the high extinction coefficient (*ε* = 9.1 × 10^3^ M^−1^ cm^−1^ for 1 and *ε* = 1.1 × 10^4^ M^−1^ cm^−1^ for 2).^30^ The weak absorption tails in both 1 and 2 are mainly attributed to ^3^MLCT absorption.^31^

### Luminescence

**Fig. 3:**
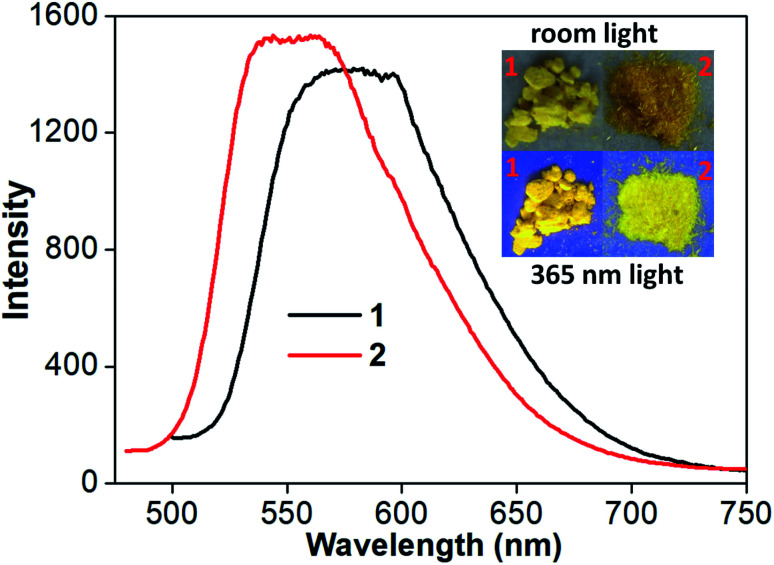
Solid-state luminescence spectra of 1 and 2 (*λ*_ex_ = 398 nm).

At room temperature, complexes 1 and 2 in CH_2_Cl_2_ are nonluminescent although ligand pbdtim in CH_2_Cl_2_ shows strong fluorescence with an emission at 409 nm [quantum yield *Φ* = 89.9%, and emission lifetimes *τ*_1_ = 1.1 ns (85.5%), *τ*_2_ = 3.4 ns (14.5%)] (Fig. S14 and Table S4†). The luminescence quenching of 1 and 2 in CH_2_Cl_2_ could be due to molecular vibration and/or some nonradiative processes.^32^ It should be noted that the crystalline samples of both 1 and 2 show significant yellow emission (Fig. 3 and Table S5†), with a broad emission peak around 579 nm for 1, and around 551 nm for 2. The quantum yields and emission lifetimes are *Φ* = 7.2%, *τ*_1_ = 7789 ns (62.9%), *τ*_2_ = 3008 ns (35.5%), *τ*_3_ = 333 ns (1.6%) for 1, and *Φ* = 6.8%, *τ*_1_ = 4362 ns (86.9%), *τ*_2_ = 845 ns (12.5%), *τ*_3_ = 59 ns (0.6%) for 2. These luminescence data (*i.e. λ*_em_, *Φ* and *τ*) indicate that the emissions of 1 and 2 in solid state have typical phosphorescence character,^5^ significantly differing from the fluorescence emission at 393 nm from the solid-state sample of pbdtmi (see *Φ* = 1.0%, *τ*_1_ = 5.2 ns (80.6%), *τ*_2_ = 0.5 ns (19.4%) in Table S5†). It should be noted that the emission bands of both 1 and 2 are very broad (Fig. 3), indicating typical ^3^MLCT character. The phosphorescence emissions from the crystalline samples of 1 and 2 can be assigned to the inter-molecular π⋯π stacking interactions (Fig. 2 and S6†) in the resultant supramolecular dimers. This can be further confirmed by the following grinding and crystallization experiments.

**Fig. 4:**
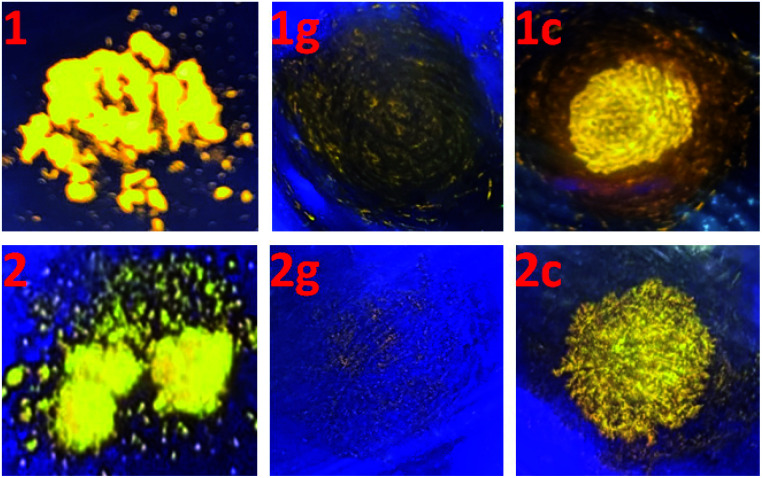
Emission colors of complexes 1 and 2 (left), their corresponding ground samples 1g and 2g (middle), and the toluene crystallization samples 1c and 2c (right) at room temperature, under a lamp with 365 nm wavelength light.

It is well known that intraligand (IL) charge transfer can result in luminescence quenching in some metal complexes.^33^ The crystalline samples of 1 and 2 are luminescent, mainly due to the fact that their dimer structures invalidate the intraligand (IL) charge transfer from pyridine–imidazole moiety to thiophene unit in ligand pbdtmi.^34^ These dimer structures can be destructed by grinding treatment, thus the intraligand (IL) charge transfer can fluently occur in the grinding samples 1g and 2g, and leading to luminescence quenching (Fig. 4). Considering that toluene can promote the crystallization of complexes 1 and 2 (see synthesis details), we add a drop of toluene into a grinding sample (1g or 2g). The resultant crystallization samples 1c and 2c show yellow emissions, indicating the recovery of the initial supramolecular dimer structures. Clearly, the phosphorescence of 1 and 2 can be reversibly switched between on-state and off-state by using grinding and crystallization processes to modulate the supramolecular dimer in these complexes, which is clearly different from the widely reported grinding-induced luminescence switching between two kinds of emission colors in some reported Pt(ii) complexes, for example, a series of pinene-containing Pt(N^N^C)Cl complexes.^35^ To our knowledge, complexes 1 and 2 are two exclusive dithienylethene-based Pt(ii) complexes showing reversible grinding- and crystallization-induced phosphorescence switching.

Moreover, the crystalline sample of complex 1 reveals longer emission length than that of complex 2 (emission wavelength: 579 nm for 1, and 551 nm for 2), although there are two draw-electron F substituent groups in the molecular structure of 1. This can be due to relatively strong π⋯π stacking interaction in complex 1 [see Fig. 2 and S6,† centroid–centroid distance between dfppy^−^/ppy^−^ ligand and pbdtmi ligand: 3.544(1) Å in 1, and 3.595(1) Å in 2].

### Photochromism study

**Fig. 5:**
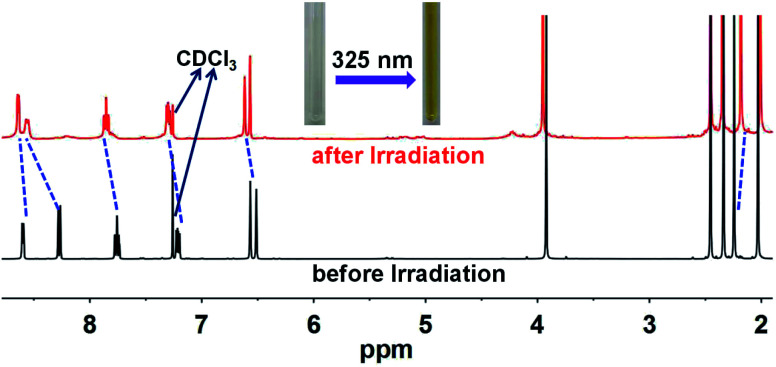
^1^H NMR spectra of pbdtmi before and after irradiation, where the dotted lines indicate the shifts of some signals.

Ligand pbdtmi shows photochromic behavior in solution at room temperature, which is confirmed by its ^1^H NMR spectra and UV-vis absorption spectra (Fig. 5, S1, S2, S12 and S13†). Before and after irradiation with 325 nm light, the ^1^H NMR spectra of pbdtmi in CDCl_3_ show clear changes in peaks of aromatic H atoms and methyl groups belonging to two thiophene groups (Fig. 5). The irradiation resulted in downfield shift for the chemical shifts of the corresponding aromatic protons, while upfield shift for the chemical shifts of the methyl groups at two thiophene groups. Moreover, upon irradiation, the CDCl_3_ solution of pbdtmi changed its color from colorless to brown (Fig. 5). These changes indicate that ligand pbdtmi has photochromic property, which is further confirmed by the absorption-spectrum change of pbdtmi in CH_2_Cl_2_ upon irradiation with 325 nm light. Under the irradiation with 325 nm light, the original strong absorption band at ∼320 nm shows slight decrease in intensity, and forms a new broad absorption band around 595 nm (Fig. S12†). This indicates the structural transition of pbdtmi from open form to closed form. This new absorption band could slowly disappear under the condition of being irradiated with 600 nm light or staying in the dark at room temperature (Fig. S13†), indicating that the structural transition of pbdtmi from closed form to open form.

Compared to free ligand pbdtmim, neither 1 nor 2 reveals obvious change in UV-vis absorption spectrum upon irradiation with 365 nm light (Fig. S10 and S11†), indicating that the two complexes have no photochromic property. The absence of photochromism in both 1 and 2 could be mainly due to the following two factors.^31^ The one is that the UV excitation energy is transferred to {Pt(dfppy/ppy)}^+^ unit, resulting in the complete absence of photocyclization process of the ligand pbdtmi. The other one is that the coordination of pbdtmi to {Pt(dfppy/ppy)}^+^ unit leads to a crowded space which hinders the ligand pbdtmi from taking part in the photocyclization reaction.

## Conclusions

In summary, two dithienylethene-based Pt(ii) complexes [Pt(dfppy)(pbdtmi)]PF_6_ (1) and [Pt(ppy)(pbdtmi)]PF_6_ (2) have been designed and synthesized. Their crystal structures indicate that two neighboring [Pt(dfppy/ppy)(pbdtmi)]^+^ cations connect each other through π⋯π stacking interaction, thus forming supramolecular dimer structure (Fig. 2 and S7†). Both 1 and 2 are nonluminescent in CH_2_Cl_2_, but their crystalline state samples reveal strong phosphorescence (emission peak: around 579 nm for 1, and 551 nm for 2), mainly due to their supramolecular dimer structures. Interestingly, these crystalline samples show reversible phosphorescence switching between on-state and off-state upon grinding and crystallization with toluene, because of the grinding-induced destruction and crystallization-induced formation of supramolecular dimer structure. Our work indicates that the construction of supramolecular dimer structure is one of feasible approaches to design and synthesize smart luminescent coordination complexes, which can respond to external stimuli (*e.g.* grinding, crystallization).

## Experimental

### Materials and methods

Compounds [Pt(dfppy)(SEt_2_)Cl] and [Pt(ppy)(SEt_2_)Cl] were prepared according to the literatures.^36^ All other reagents were commercially available and used without further purification. Elemental analyses were performed on a PerkinElmer 240C elemental analyzer. IR spectra were obtained as KBr disks on a VECTOR 22 spectrometer. The ^1^H NMR spectra were recorded at room temperature with a 400 MHz BRUKER spectrometer. UV-vis absorption spectra were measured on a Cary 100 spectrophotometer. Luminescence spectra were measured using a Hitachi F-4600 fluorescence spectrometer. The luminescence lifetimes were measured at room temperature on a HORIBA FL-3 Spectrofluorometer with a 370 nm LED pulsed from a NanoLED resource. The luminescence quantum yield of pbdtmi in CH_2_Cl_2_ solution was measured by a relative method by comparison with a standard, a solution of quinine sulfate in 0.5 M H_2_SO_4_ (*Φ* = 54.6%, *λ*_ex_ = 366 nm).^37^ The quantum yields of the crystalline samples of 1 and 2 were measured at room temperature on a Horiba FL-3 spectrofluorometer.

### Synthesis of 2-pyridyl-4,5-bis(2,5-dimethyl(3-thienyl))-1-methyl-imidazole (pbdtmi)

A mixture of 2-pyridyl-4,5-bis(2,5-dimethyl(3-thienyl))-1*H*-imidazole (0.413 mmol, 0.1510 g) and K_2_CO_3_ (1.65 mmol, 0.2284 g) was stirred in DMF (5 mL) at room temperature for 45 minutes. Then a solution of CH_3_I (0.413 mmol, 26.4 μL) in DMF (4 mL) was added dropwise, and the reaction mixture was stirred for additional 20 hours. The reaction mixture was mixed with H_2_O (15 mL) and extracted with ethyl acetate (30 mL × 3). The combined ethyl acetate solution was washed with saturated NaCl aqueous solution (20 mL × 10) to remove DMF, dried with MgSO_4_, filtered, and then evaporated, obtaining a white solid with a yield of 147 mg (94% based on 2-pyridyl-4,5-bis(2,5-dimethyl(3-thienyl))-1*H*-imidazole). Anal. calcd for C_21_H_21_N_3_S_2_: C, 66.46; H, 5.58, N, 11.07. Found: C, 66.61; H, 5.79, N, 11.23. IR (KBr, cm^−1^): 3537(w), 3046(w), 2913(w), 2852(w), 1589(s), 1565(w), 1512(m), 1461(s), 1421(w), 1383(w), 1347(w), 1278(w), 1141(s), 1070(w), 1039(w), 990(w), 952(w), 841(s), 785(w), 737(w), 704(m), 623(w), 495(m). ^1^H NMR (400 MHz, CDCl_3_), *δ* (ppm): 2.03–2.45 (4s, 12H from four –CH_3_ groups attached to two thiophene rings), 3.92 (s, 3H from a –CH_3_ group attached to the imidazole unit in pbdtmi), 6.51 and 6.57 (2s, 2H from two thiophene rings), 7.22 (t, *J* = 6.2, 1H), 7.76 (t, *J* = 8.8, 1H), 8.28 (d, *J* = 8.0, 1H) and 8.60 (d, *J* = 4.8, 1H) (7.22 and 7.76–8.60, total 4H from a pyridyl unit in pbdtmi).

### Synthesis of [Pt(dfppy)(pbdtmi)]PF_6_ (1)

To a solution of pbdtmi (0.1 mmol, 0.0380 g) and [Pt(dfppy)(SEt_2_)Cl] (0.1 mmol, 0.0440 g) in CH_2_Cl_2_ (6 mL) was added AgPF_6_ (0.1 mmol, 0.0253 g). The reaction mixture was heated in an oil bath (40 °C) for one day, and then 30 mL of CH_2_Cl_2_ was added. The mixture was filtered to remove AgCl solid. The filtrate was evaporated under vacuum, and the residue was washed with toluene, obtaining yellow crude product. The CH_2_Cl_2_–toluene solution of the crude product was allowed to slowly evaporate, forming yellow blocky crystals of complex 1 with a yield of 61 mg (67% based on [Pt(dfppy)(SEt_2_)Cl]). Anal. calcd for C_32_H_27_N_4_F_8_PS_2_Pt: C, 42.25%; H, 2.99%, N, 6.16%. Found: C, 42.31%; H, 3.17%; N, 6.30%. IR (KBr, cm^−1^): 3444(w), 1605(m), 1574(w), 1523(w), 1481(m), 1452(w), 1429(w), 1408(w), 843(s), 780(w), 751(w), 557(m). ^1^H NMR (400 MHz, CDCl_3_), *δ* (ppm): 2.01–2.46 (m, 12H from four –CH_3_ groups attached to two thiophene rings), 4.07 (s, 3H from a –CH_3_ group attached to the imidazole unit in pbdtmi), 6.41 and 6.82 (m, 5H), 7.62 (t, *J* = 7.2, 1H), 7.78 (t, *J* = 8.0, 1H), 7.99 (d, *J* = 8.4, 2H), 8.47–8.53 (d, 2H) and 9.15 (d, *J* = 5.2, 1H) (6.41–6.82 and 7.62–9.15, total 12H from ligands pbdtmi and dfpppy^−^).

### Synthesis of [Pt(ppy)(pbdtmi)]PF_6_ (2)

Compound 2 was prepared by the same method as 1, using [Pt(ppy)(SEt_2_)Cl] instead of [Pt(dfppy)(SEt_2_)Cl]. The crude product of 2 was crystallized in a CH_2_Cl_2_–toluene solution, obtaining yellow blocky crystals with a yield of 59 mg (61% based on [Ir(ppy)_2_Cl]_2_). Anal. calcd for C_39_H_37_N_4_F_6_PS_2_Pt (2·toluene): C, 48.50%; H, 3.86%, N, 5.80%. Found: C, 48.54%; H, 3.97%; N, 5.95%. IR (KBr, cm^−1^): 3444(w), 1609(m), 1521(w), 1480(m), 1447(w), 1159(w), 840(s), 755(m), 557(m). ^1^H NMR (400 MHz, CD_2_Cl_2_), *δ* (ppm): 1.94–2.47 (m, 12H from four –CH_3_ groups attached to two thiophene rings), 4.01 (s, 3H from a –CH_3_ group attached to the imidazole unit in pbdtmi), 6.46–6.63(m, 3H), 7.26–7.38 (m, 3H), 7.58 (d, *J* = 8.0, 1H), 7.65 (t, *J* = 7.4, 1H), 7.72 (d, *J* = 7.2, 1H), 7.81 (t, *J* = 8.6, 1H), 7.81 (broad peak, 1H), 8.26 (d, *J* = 8.0, 1H), 8.41 (t, *J* = 8.0, 1H) and 9.40 (d, *J* = 6.0, 1H) (6.46–9.40, total 14H from ligands pbdtmi and ppy^−^).

### X-ray crystallographic study

Single crystal of dimension 0.12 × 0.10 × 0.10 mm^3^ for 1, and 0.21 × 0.16 × 0.13 mm^3^ for 2·C_7_H_8_ (C_7_H_8_ = toluene) were used for structural determination on a Bruker SMART APEX CCD diffractometer (*λ*_MoKα_ = 0.71073 Å) for 1 at room temperature, and on a Bruker D8 Venture diffractometer (*λ*_GaKα_ = 1.34138 Å) for 2·C_7_H_8_ at 193(2) K. A hemisphere of data were collected in the *θ* range 2.274 to 27.488° for 1, and 2.529 to 53.785° for 2·C_7_H_8_ using a narrow-frame method with scan width *ω* = 0.50° for 1 and *ω* = 0.90° for 2·C_7_H_8_, and an exposure time of 10 s per frame. Numbers of observed and unique reflections are 14 076 and 7311 (*R*_int_ = 0.0465) for 1, and 49 312 and 6609 (*R*_int_ = 0.0668) for 2·C_7_H_8_, respectively. The data were integrated using the Siemens *SAINT* program,^38^ with the intensities corrected for Lorentz factor, polarization, air absorption, and absorption due to variation in the path length through the detector faceplate. Multi-scan absorption corrections were applied. The structures were solved by direct methods and refined on *F*^2^ by full matrix least squares using *SHELXTL*.^39^ All the non-hydrogen atoms were located from the Fourier maps, and were refined anisotropically. All H atoms were refined isotropically, with the isotropic vibration parameters related to the non-H atom to which they are bonded. In the structural refinements of 1 and 2·C_7_H_8_, ISOR, SIMU and SADI were used to refine disordered lattice toluene molecule, PF_6_^−^ ion and thiophene group. The crystallographic data for compounds 1 and 2·C_7_H_8_ are listed in Table S1,† and selected bond lengths and bond angles are given in Tables S2 and S3.† CCDC 2122586 and 2099083 contain the supplementary crystallographic data of 1 and 2·C_7_H_8_, respectively.

## Conflicts of interest

There are no conflicts declare.

## Supplementary Material

RA-012-D1RA07142D-s001

RA-012-D1RA07142D-s002
