# Supplementary material for: Two cyclometalated Pt(ii) complexes showing reversible phosphorescence switching due to grinding-induced destruction and crystallization-induced formation of supramolecular dimer structure
Source: RSC Adv. 2021 Dec 21;12(1):148–53. doi: 10.1039/d1ra07142d (PMC8978655; doi:10.1039/d1ra07142d)
Supplement: RA-012-D1RA07142D-s001 [file RA-012-D1RA07142D-s001.pdf]

Electronic Supplementary Material (ESI) for RSC Advances.
This journal is © The Royal Society of Chemistry 2021

Electronic Supporting Information

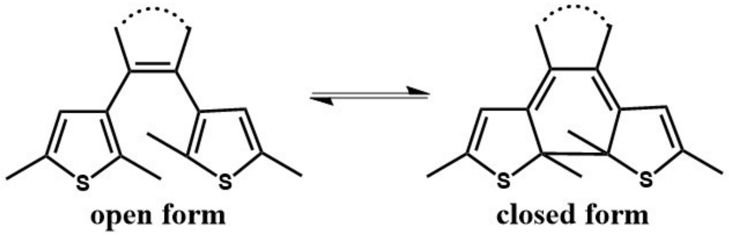

Scheme S1 Structural transition between open form and closed form for dithienylethene (DTE).

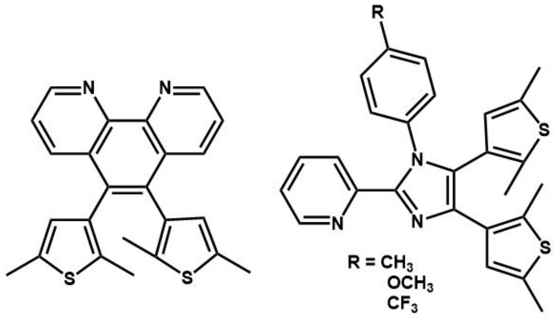

Scheme S2 The molecular structures of the reported DTE-based N^N ancillary ligands in references 23 and 25 in the main text.

Table S1 Crystallographic data and refinement for **1** and **2**·C_7H_8

	**1**	**2**·C_7H_8
Formula	$C_{32}H_{27}N_4F_8PS_2Pt$	$C_{39}H_{37}N_4F_6PS_2Pt$
M	909.75	965.90
Crystal system	Monoclinic	Monoclinic
Space group	$P2_1/n$	$P2_1/c$
T /K	296(2)	193(2)
a /Å	9.0408(5)	7.2607(3)
b /Å	17.9156(8)	27.1529(10)
c /Å	19.9292(9)	18.6030(8)
β /°	96.929(2)	99.488(2)
V/Å^3	3204.4(3)	3617.4(3)
Z	4	4
D_c /g cm^{-3}	1.886	1.774
F(000)	1776	1912
GooF on F^2	1.036	1.047
R_1, wR_2 [I>2σ(I)]^a	0.0378, 0.0758	0.0390, 0.0856
R_1, wR_2 (all data)^a	0.0601, 0.0823	0.0531, 0.0914
$(\Delta\rho)_{max}$, $(\Delta\rho)_{min}$ (e Å^{-3})	1.011, -1.266	1.029, -1.052

^{a} $R_1 = \Sigma||F_o|-|F_c||/\Sigma|F_o|$. $wR_2=[\Sigma w(F_o^2-F_c^2)^2/\Sigma w(F_o^2)^2]^{1/2}$.

Table S2 Selected bond lengths (Å) and bond angles (°) for **1**

Pt1-C1	2.002(5)	Pt1-N3	2.111(4)
Pt1-N1	2.010(4)	N3-C17	1.346(6)
Pt1-N2	2.040(4)	N4-C17	1.358(6)
C1-Pt1-N1	79.75(19)	C7-N1-Pt1	117.1(3)
C1-Pt1-N2	101.30(18)	C12-N2-Pt1	127.2(4)
N1-Pt1-N2	178.32(18)	C16-N2-Pt1	115.0(3)
C1-Pt1-N3	176.99(17)	C18-N3-Pt1	142.6(3)
N1-Pt1-N3	101.14(16)	C17-N3-Pt1	109.9(3)
N2-Pt1-N3	77.88(15)	C2-C1-Pt1	130.5(4)
C11-N1-Pt1	124.1(3)	C6-C1-Pt1	112.2(4)

Table S3 Selected bond lengths (Å) and bond angles (°) for **2**·C_7H_8

Pt1-C1	2.008(5)	Pt1-N3	2.124(4)
Pt1-N1	2.011(5)	N3-C17	1.345(7)
Pt1-N2	2.050(4)	N4-C17	1.361(7)
C1-Pt1-N1	79.8(2)	C7-N1-Pt1	115.8(4)
C1-Pt1-N2	101.0(2)	C12-N2-Pt1	126.3(4)
N1-Pt1-N2	178.73(18)	C16-N2-Pt1	115.4(3)
C1-Pt1-N3	177.02(19)	C17-N3-Pt1	111.0(4)
N1-Pt1-N3	101.76(18)	C18-N3-Pt1	142.0(4)
N2-Pt1-N3	77.39(17)	C6-C1-Pt1	112.5(4)
C11-N1-Pt1	125.5(4)	C2-C1-Pt1	131.6(4)

Table S4 Photophysical properties of **1**, **2** and pbdtmi in CH_2Cl_2 at room temperature.

Compound	Medium	λ_{abs} (nm)	λ_{em} (nm)	Lifetime (ns)	quantum yield
**1**	CH_2Cl_2 (298 K)	306, 359 and a tail to 448	-	-	-
**2**	CH_2Cl_2 (298 K)	310, 363 and a tail to 456	-	-	-
pbdtmi	CH_2Cl_2 (298 K)	320	409	1.1 (85.5%), 3.4 (14.5%)	89.9%

Table S5 Luminescence data of **1**, **2** and pbdtmi in solid state at room temperature.

Compound	λ_{em} (nm)	Lifetime (ns)	quantum yield
**1**	579	7789 (62.9%),<br>3008 (35.5%),<br>333 (1.6%)	7.2%
**2**	551	4362 (86.9%),<br>845 (12.5%),<br>59 (0.6%)	6.8%
pbdtmi	393	5.2 (80.6%),<br>0.5 (19.4%)	1.0%

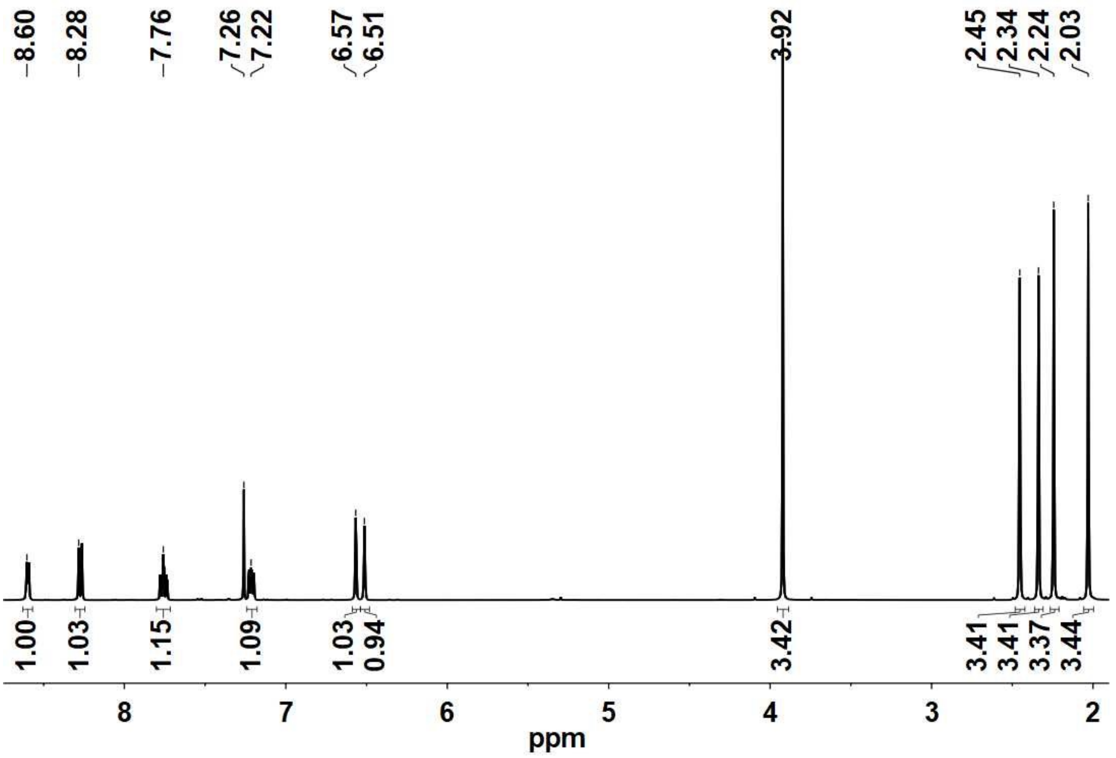

Fig. S1 The ^{1}H NMR of pbdtmi in $CDCl_3$.

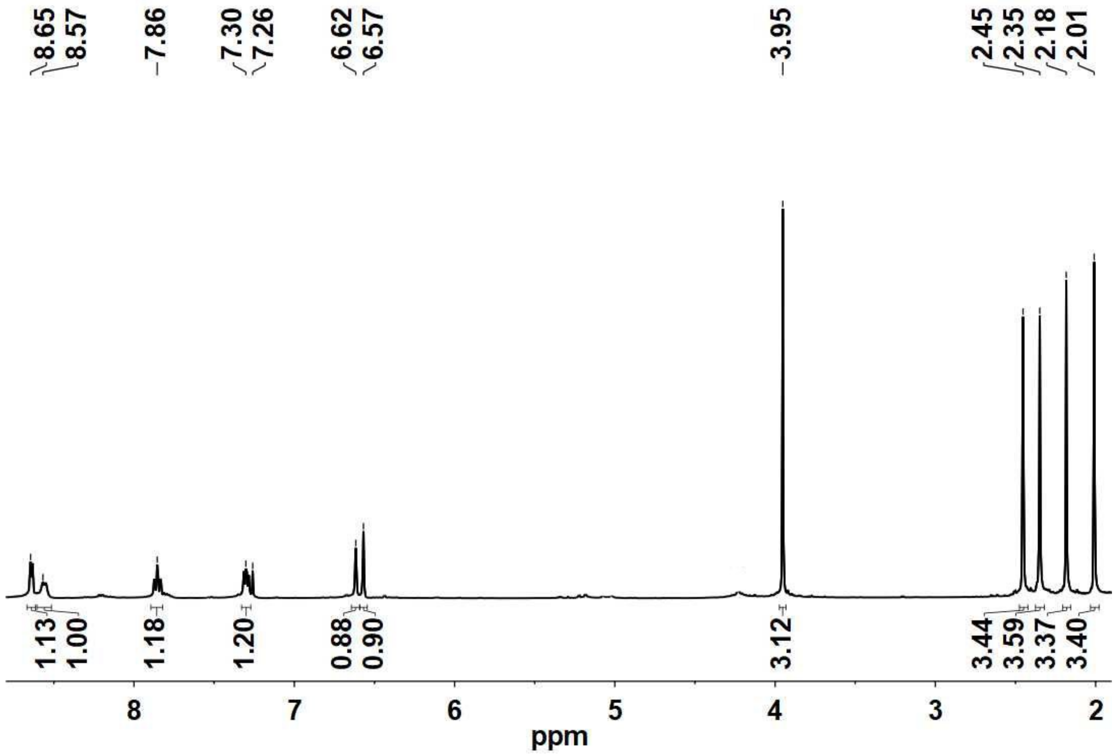

Fig. S2 The ^{1}H NMR of pbdtmi in $CDCl_3$ after irradiation for 30 min under 365 nm light [δ (ppm): 2.01-2.45 (4s, 12H from four -CH_3 groups attached to two thiophene rings), 3.95 (s, 3H from a -CH_3 group attached to the imidazole unit in pbdtmi), 6.57 and 6.62 (2s, 2H from two thiophene rings), 7.30 (t, J = 6.2, 1H), 7.86 (t, J = 7.8, 1H), 8.57 (d, J = 6.8, 1H) and 8.65 (d, J = 4.8, 1H) (7.30-8.65, total 4H from a pyridyl unit in pbdtmi).]

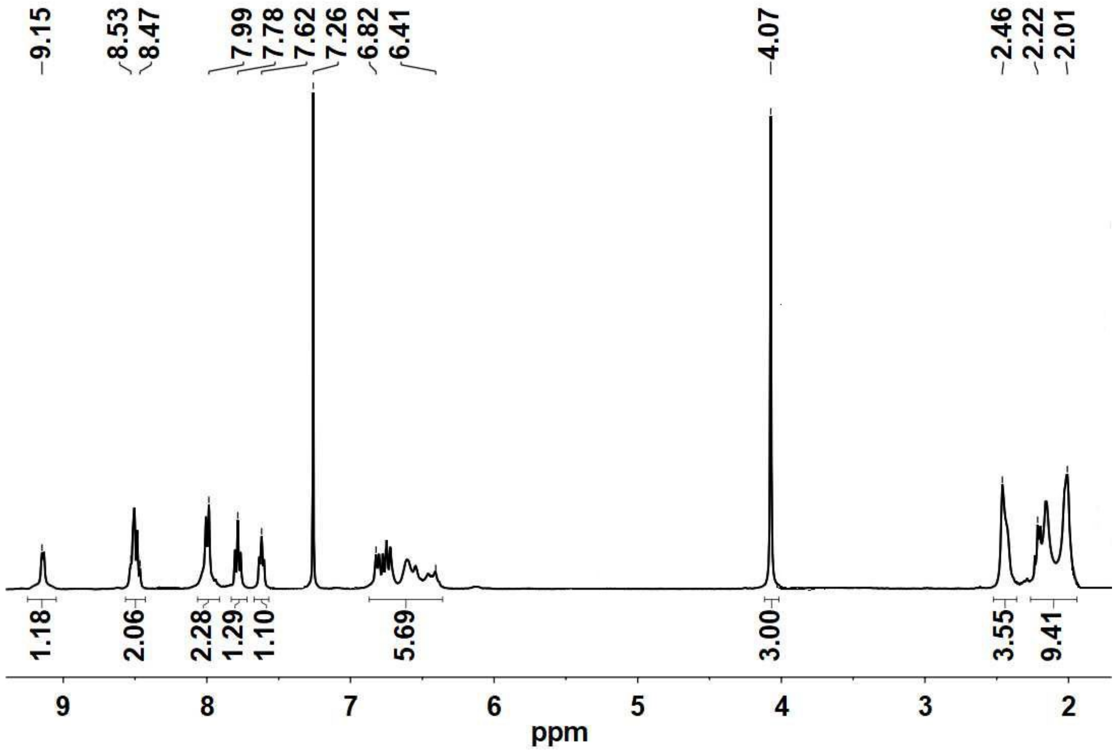

Fig. S3 ^{1}H NMR of **1** in $CDCl_3$

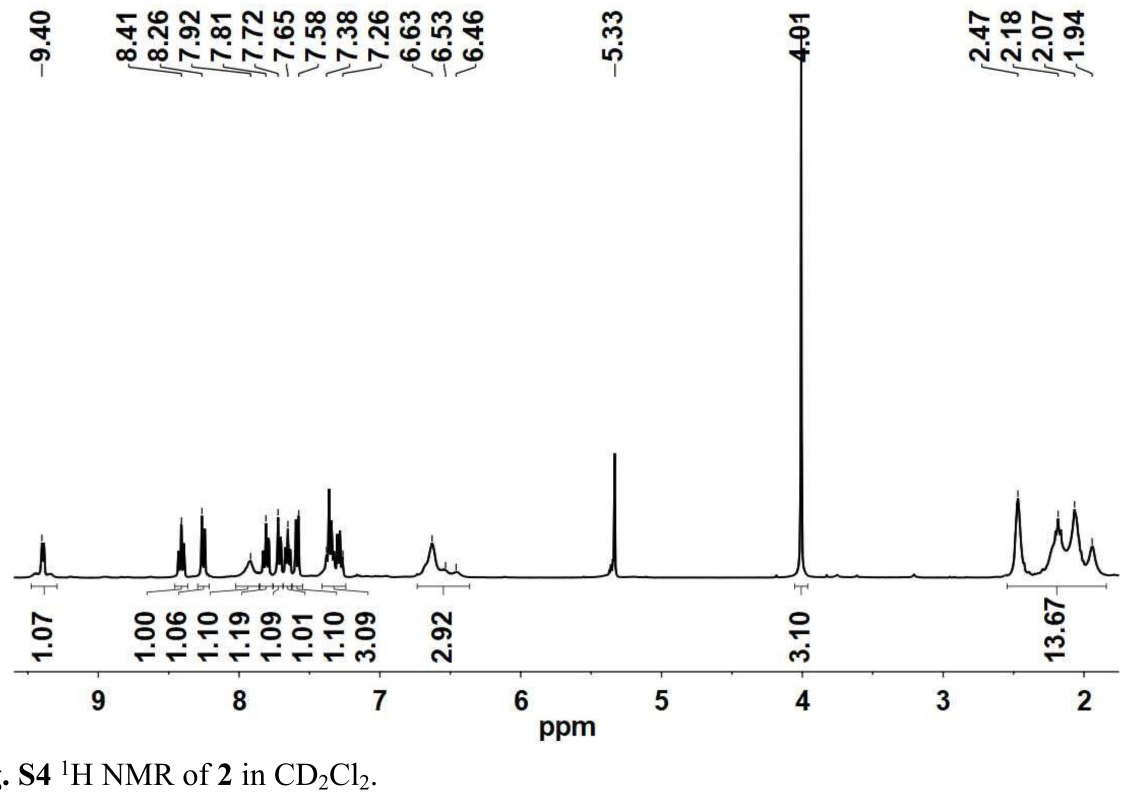

Fig. S4 ^{1}H NMR of **2** in CD_2Cl_2.

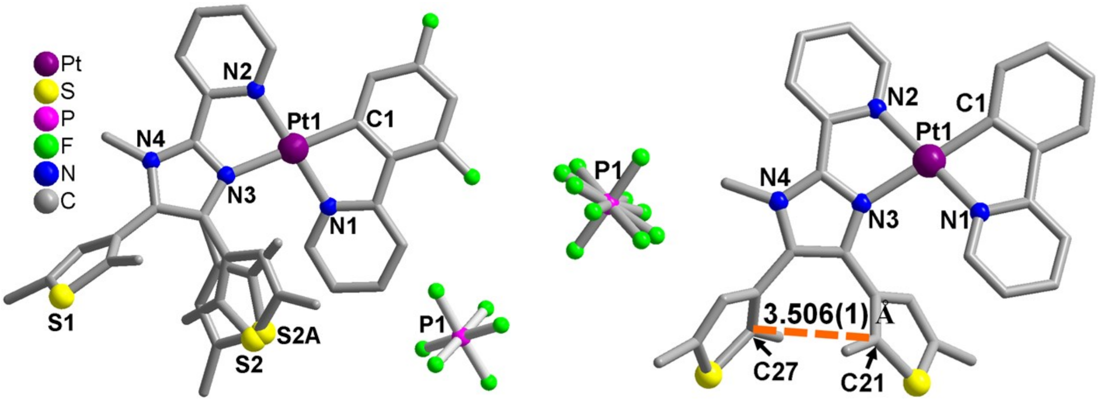

Fig. S5 Molecular structures of **1** (left) and **2** (right). All H atoms attached to carbon atoms are omitted for clarity. One of two thiophene groups in **1** and the PF_6^- ion in **2** are disordered.

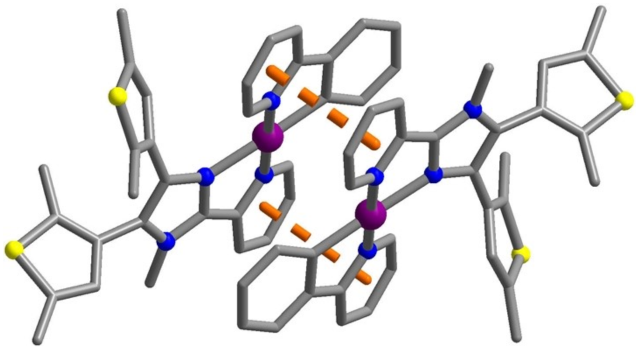

Fig. S6 Supramolecular dimer structure in complex **2**·C_7H_8.

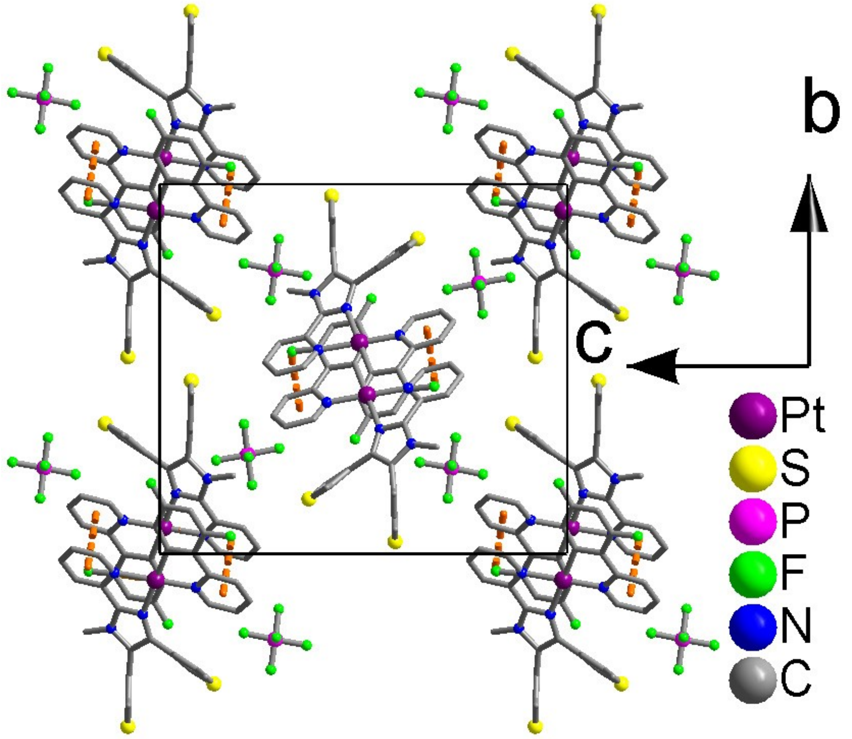

Fig. S7 Packing structure of **1**. The disordered states of thiophene groups in this Fig. have not been shown for clarity.

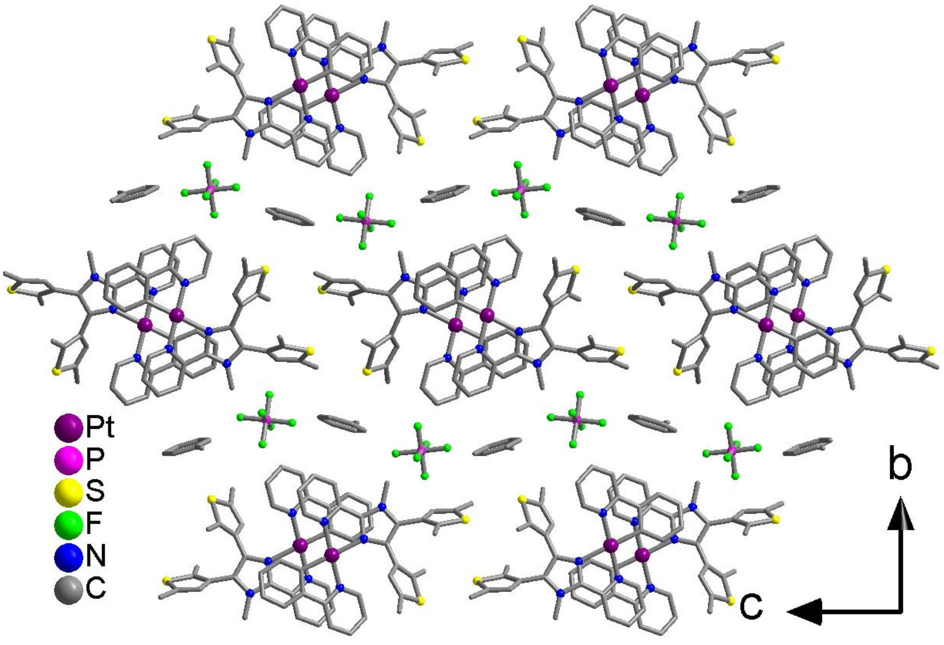

Fig. S8 Packing structure of **2**·C_7H_8. The toluene molecules and PF_6^- ions in this structure are disordered. However, their disordered states have not been shown in this figure for clarity.

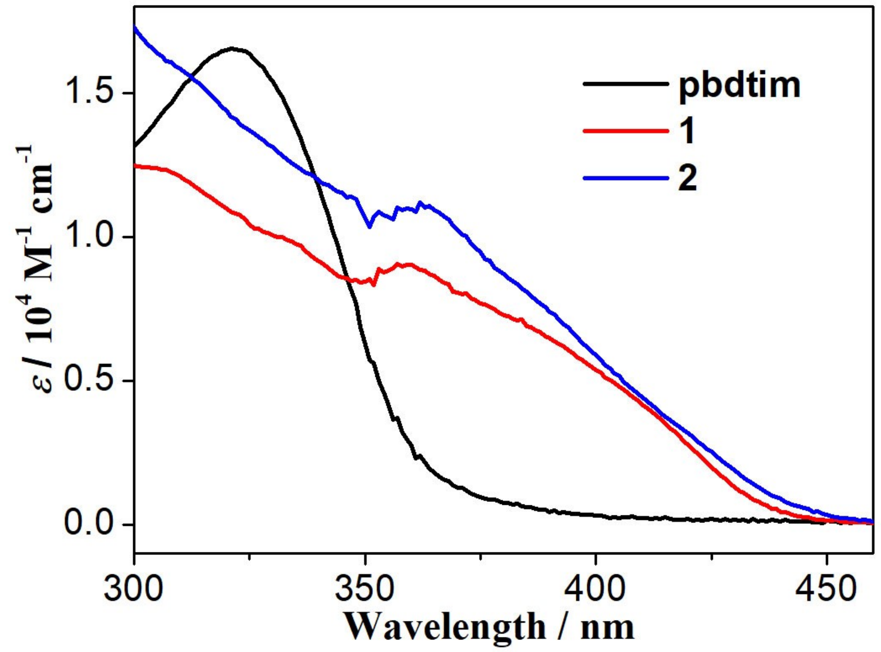

Fig. S9 UV-v is spectra of pbdtmi, **1** and **2** in CH_2Cl_2.

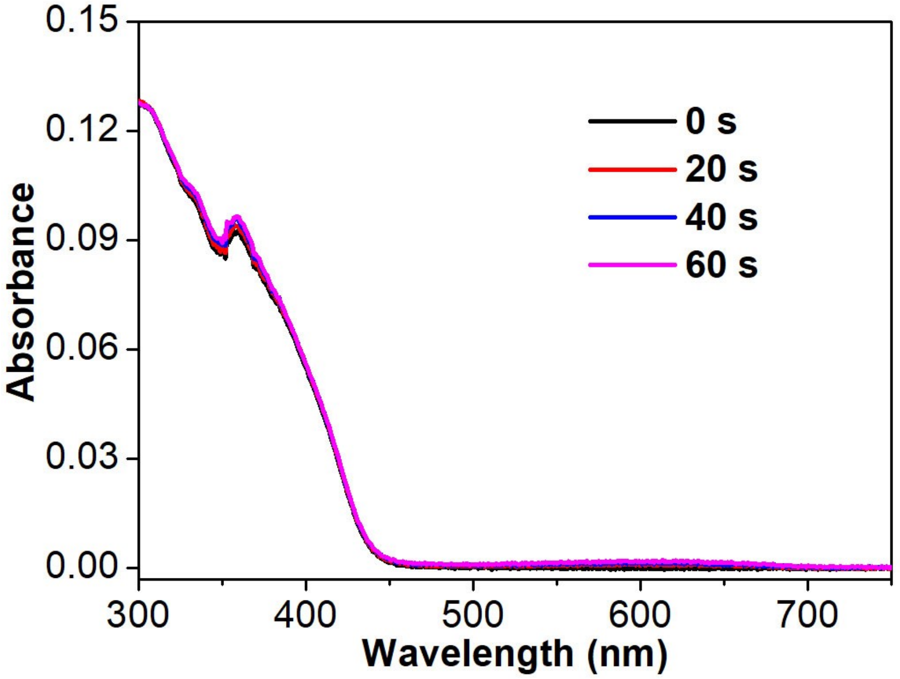

Fig. S10 Absorption spectra changes of **1** in CH_2Cl_2 solution ($c = 1.0 \times 10^{-5}$ M) upon UV irradiation ($\lambda = 365$ nm) for 0–60 seconds.

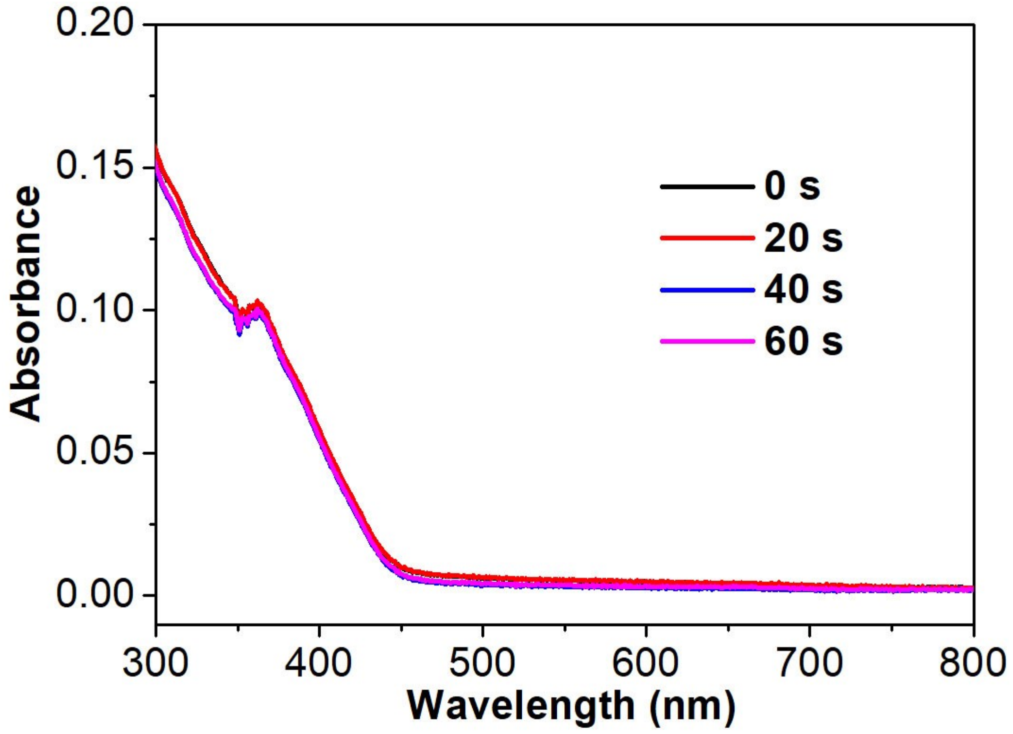

Fig. S11 Absorption spectra changes of **2** in CH_2Cl_2 solution (c = 1.0 × 10^{-5} M) upon UV irradiation (λ = 365 nm) for 0–60 seconds.

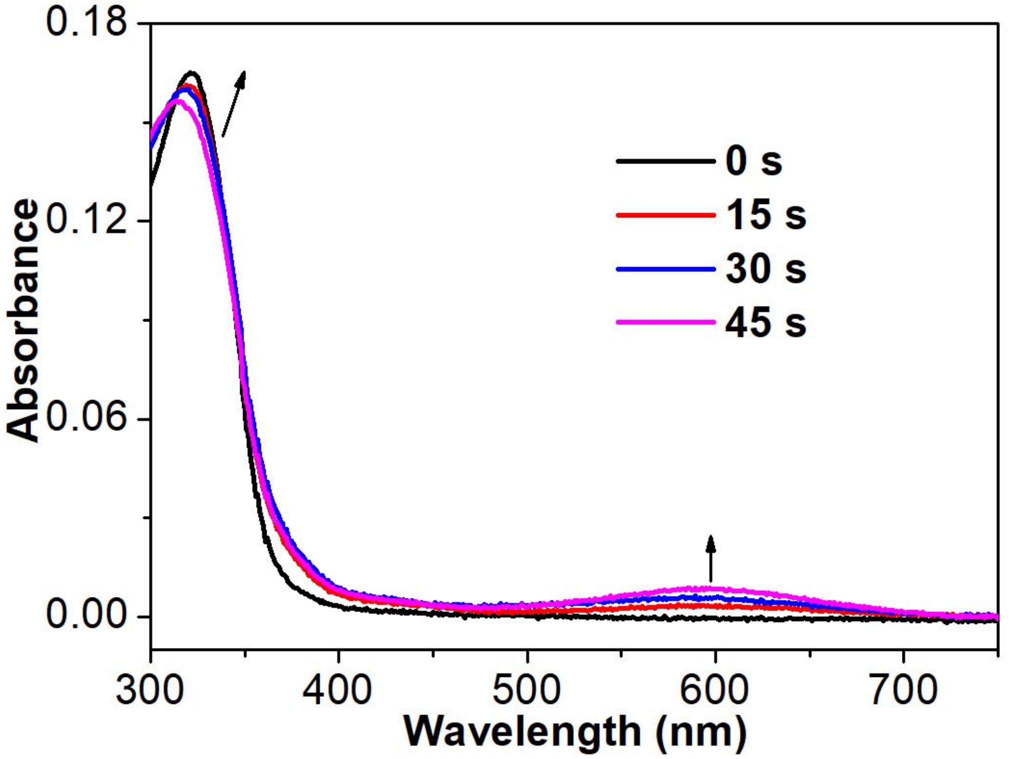

Fig. S12 Absorption spectra changes of pbdtmi in CH_2Cl_2 solution (c = 1.0 × 10^{-5} M) upon UV irradiation (λ = 325 nm) for 0–45 seconds.

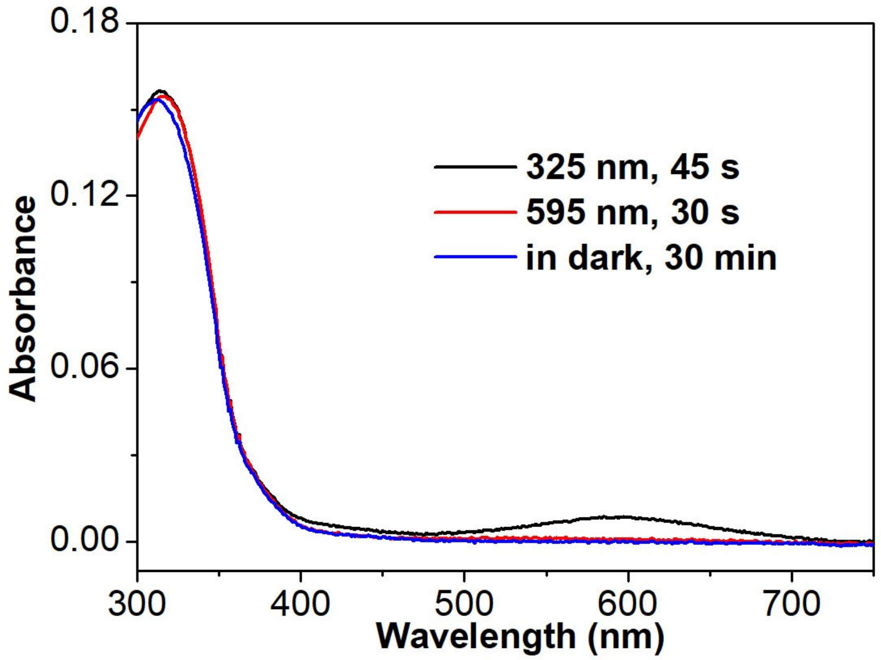

Fig. S13 Black line: irradiating (λ = 325 nm) the CH_2Cl_2 solution of pbdtmi in CH_2Cl_2 for 45 seconds; red line: irradiating (λ = 595 nm) the solution corresponding to black line for 30 seconds; blue line: placing the solution corresponding to black line in the dark for 30 minutes.

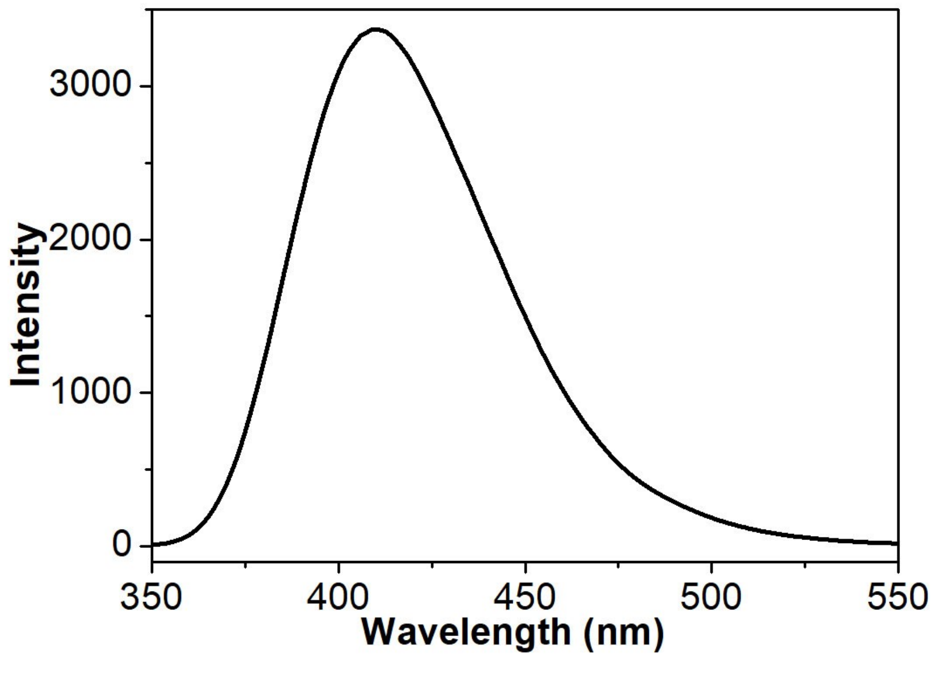

Fig. S14 Luminescence spectrum of pbdtmi in CH_2Cl_2 solution (c = 1.0 × 10^{-4} M, λ_{ex} = 336 nm).
